# Tandem Catalysis of an Aldol-‘Click’ Reaction System within a Molecular Hydrogel

**DOI:** 10.3390/molecules21060744

**Published:** 2016-06-08

**Authors:** Marco Araújo, Iván Muñoz Capdevila, Santiago Díaz-Oltra, Beatriu Escuder

**Affiliations:** Departament de Química Inorgànica i Orgànica, Universitat Jaume I, 12071 Castelló, Spain; cerqueir@uji.es (M.A.); munozi@uji.es (I.M.C.); sdiaz@uji.es (Q.D.-O.)

**Keywords:** aldol addition, click reaction, tandem catalysis, supramolecular gels, multicomponent system

## Abstract

A heterogeneous supramolecular catalytic system for multicomponent aldol-‘click’ reactions is reported. The copper(I) metallohydrogel functionalized with a phenyltriazole fragment was able to catalyze the multicomponent reaction between phenylacetylene, *p*-nitrobenzaldehyde, and an azide containing a ketone moiety, obtaining the corresponding aldol products in good yields. A possible mechanistic pathway responsible for this unexpected catalytic behavior has been proposed.

## 1. Introduction

Enzymes, the catalysts used by living organisms, are a clear example of nature efficiency. These organisms are responsible to perform diverse chemical transformations with high selectivity and specifity, being able to produce structurally complex compounds through multienzimatic cascade reactions [1]. By contrast, in classical synthetic chemistry, individual transformations are conducted as stepwise processes, punctuated by the purification and isolation of intermediates at each stage of the sequence. In an attempt to mimic different aspects of synthetic strategies that operate in biological systems, chemists have been challenged to design artificial systems that simulate the enzymatic ability of performing multistep reactions [2]. Supramolecular systems such as multicomponent transition metal materials formed by self-assembly of programmed ligand building blocks, molecular gels, metal-organic frameworks, cyclodextrin derived compounds, or dendrimers have been widely investigated as biomimetic systems [3,4,5,6,7,8,9,10]. As a result of a growing interest in mimicking nature processes, much attention has been directed to “one-pot” processes involving multiple catalytic transformations followed by a single work up [11]. “One-pot” tandem reactions, where multiple catalysts and reagents combined in a single reaction vessel embark in a sequence of precisely staged catalytic steps have become highly attractive [12]. Depending on the number of catalytic species or whether there is intervention in the reaction system, tandem catalysis can be subcategorized in three different categories: orthogonal, auto-tandem, or assisted tandem catalysis [13]. In orthogonal tandem catalysis sequential catalytic processes occur through two or more functionally distinct, and preferably non-interfering catalytic cycles whereas, in auto-tandem or assisted tandem processes, the catalysis occurs in the presence of a unique catalyst [14]. The main feature that distinguishes between these last two processes is the spontaneity of the catalytic processes. While auto-tandem catalysis involves two or more mechanistically distinct catalysis promoted by a single catalyst precursor, where both cycles occur spontaneously by cooperative interaction of several species (catalyst, substrate, additional reagents if required) present from the outset of the reaction, assisted tandem catalysis requires deliberate intervention in the reaction system to switch between two catalytic mechanisms [13].

From the synthetic chemistry point of view, ideal reaction strategies for the preparation of structurally complex substances should involve sequences in which stereocontrolled formation of multiple carbon-carbon bonds occur in a single step starting from simple and readily available materials [15]. In this field, aldol addition reactions are among the transformations that have greatly simplified the construction of asymmetric C-C bonds, being particularly important both in complex molecule synthesis and in the preparation of optically active small molecule building blocks [16]. These reactions are usually catalyzed by l-proline, following a mechanistic pathway which involves the formation of an intermediary enamine [17,18]. Indeed, supramolecular catalytic gels functionalized with l-proline fragments have been reported as efficient catalysts for aldol addition reactions [19,20].

Several tandem or multicomponent systems comprising combinations of aldol reactions with others, such as aldol-allylation [21], 1,4-addition-aldol [22,23], Michael-aldol [24], or amination-aldol [25] have been investigated for the synthesis of natural products or respective building blocks. On the other hand, in the field of ‘click’ chemistry such systems are scarce. ‘Click’ reactions are a set of powerful, highly reliable, versatile, and selective reactions that quickly generate new molecules by “clicking” small units together through heteroatom links (C-X-C) and has become the preferred strategy for the development of new compounds with desired property profiles [26]. Between all the reactions that can afford a ‘click’ status, the copper(I) catalyzed Huisgen 1,3-dipolar cycloaddition of alkynes and azides (CuAAC) is regarded as the premier example of a ‘click’ reaction, particularly due to the mild reaction conditions and regioselectivity, affording exclusively 1,4-regioisomers [27,28,29]. Molecular triazole functionalized gels have been recently reported as efficient heterogeneous supramolecular catalysts for the Huisgen 1,3-azide alkyne cycloaddition [30]. Particularly for CuAAC reaction, between the very few examples of multicomponent systems found in literature, the “one-pot” synthesis of 1,4-disubstituted 1,2,3-triazoles starting from α-bromoketone is considered one of the most practical and useful, since terminal acetylene reacts with the *in situ* generated azide, providing a safer synthetic procedure [31,32]. A clear example of a tandem ‘click’ reaction is the tandem enantioselective biocatalytic epoxide ring opening and [3+2] azide-alkyne cycloaddition, which occurs in the presence of halohydrin dehalogenase and the traditional catalytic mixture copper(II) sulphate/sodium ascorbate [33]. However, very little work has been done on matching together in a “one-pot” system aldol and ‘click’ reactions, which are nowadays highly important in the field of pharmaceutical and materials industries [34,35]. Joining together two reactions in a “one-pot” system often results in time and economy-efficient greener synthetic processes, which are highly attractive in the industrial point of view.

Having this in mind, our investigation was focused on joining these two reactions in an auto-tandem catalytic ‘click’/aldol system. In a green and sustainable chemistry perspective, water was used as the preferred solvent to carry out the multicomponent reaction. The catalytic performance of the self-assembled triazole functionalized metallogel Cu(I)-**PhTzVal_3_**, particularly suitable for the catalysis of ‘click’ reactions, was evaluated in the three-component system and a possible catalytic mechanistic pathway has been proposed. 

## 2. Results and Discussion

### 2.1. Design and Synthesis

**Figure 1:**
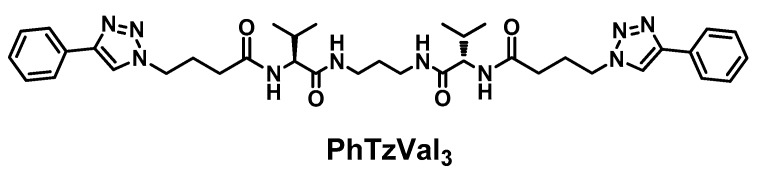
Structure of the **PhTzVal_3_** gelator.

The bolaamphiphilic l-valine derived gelator is composed by a central diaminopropane core linked to a l-valine fragment in each side and functionalized with a phenyltriazole moiety separated by a four carbon alkyl chain. The gelator has in its structure four amide bonds that, together with the aromatic rings, could behave as gelation motifs, promoting the self-assembly of the compound (Figure 1).

The synthesis of the **PhTzVal_3_** gelator involved the attachement of the activated ester of the bromobutyric acid to the three carbon alkylidendiamine [36] by simple peptide coupling, followed by a nucleophilic substitution of the halide with an azide and consequent coupling with the commercially available phenylacetylene through a copper(I) catalyzed Huisgen 1,3-diplolar cycloaddition (see Supplementary Materials).

### 2.2. Self-Assembly Studies

The **PhTzVal_3_** gelator was able to aggregate in water at concentrations of 5.7 mM, returning a swollen precipitate that can flow along the solution. The gelation procedure consisted of heating the gelator until complete dissolution, followed by 20 s of sonication. Typical thermotropic and viscoelastic properties such as gel-to-sol transitions or minimum gelation concentration have not been investigated since it was impossible to obtain a gel that did not flow upon inversion of the test tube. Unlike most metallogels described in literature [37,38,39,40,41,42], the **PhTzVal_3_** gelator was able to aggregate with and without the presence of the copper salt CuBr, discarding a metal-induced self-assembly process. Moreover, the gelator **PhTzVal_3_** exhibited an appreciable copper loading capacity, being able to coordinate 71% CuBr initially present in solution, as determined by ICP-MS. 

**Figure 2:**
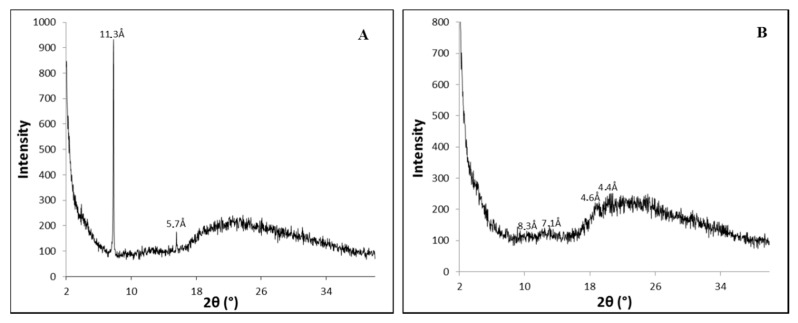
Diffractograms obtained for the hydrogels **PhTzVal_3_** (**A**) and Cu(I)-**PhTzVal_3_** (**B**).

It is thought that the **PhTzVal_3_** molecules aggregate in such a way that the triazole fragments coordinate copper(I) in a tetrahedral morphology, resembling the well-described TBTA complexes [43,44]. Thus, a possible interference of the metal on the self-assembly of the gelator was investigated by wide-angle X-ray diffraction (WAXD) (Figure 2).

The obtained diffractograms suggest that the gelator **PhTzVal_3_** aggregates in a semi-amorphous structure both in the presence and absence of the copper salt CuBr. It is curious to observe that, in the presence of copper, it seems that the gelator acquires an even more unorganized structure (Figure 2B). 

**Figure 3:**
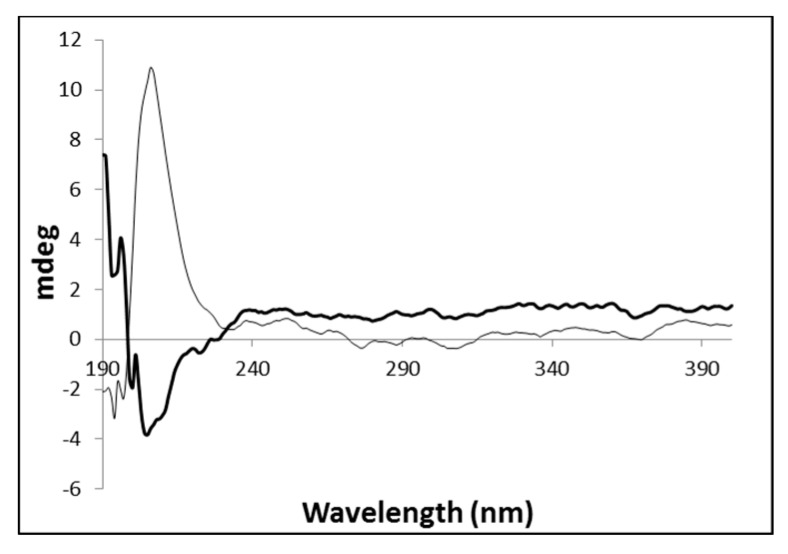
Circular dichroism spectra obtained for the hydrogels **PhTzVal_3_** (**grey**) and Cu(I)-**PhTzVal_3_** (**black**).

The native gel and the corresponding metallogel were also analyzed by circular dichroism spectroscopy to get a deeper insight on the influence of the metal on the final structural arrangement of the gel (Figure 3). This technique provides information on the conformational arrangement of self-assembled structures [45].

The native gel **PhTzVal_3_** exhibits a strong positive signal around 207 nm, which is inverted in the presence of the copper salt. This might suggest that during the self-assembly process, the coordination of the copper(I) with the triazole fragments of the gelator molecules provide an inversion in the final conformational orientation of the gel, probably due to crosslinking between the metal and the gelator molecules.

**Figure 4:**
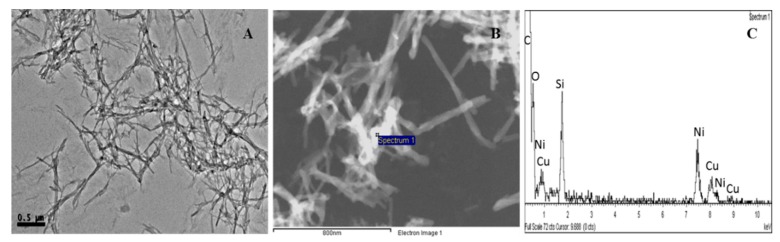
TEM analysis of the metallogel Cu(I)-**PhTzVal_3_** (**A**); STEM analysis of Cu(I)-**PhTzVal_3_** (**B**) and corresponding EDS spectrum (**C**).

The influence of the metal on the morphology of the hydrogel aggregates was investigated by Transmission Electron Microscopy (Figure 4).

The Cu(I)-**PhTzVal_3_** metallogel exhibits an entangled crosslinked network of long and thin fibers. The presence of the metal allowed the acquisition of STEM pictures without staining the samples. The coordination of copper(I) to the gel resulted in the formation of an extended fibrous network exhibiting a wide copper(I) distribution along the fibers, as evidenced by the white contrast of the metallogel network (Figure 4B). The presence of copper was further confirmed by EDS analysis, where only carbon, oxygen, copper, and nickel were detected, this last one arising from the sample grid. The absence of a 1:1 Cu:Br ratio in the EDS spectrum supports the successful coordination of the metal to the pheyltriazole gel, resulting in the formation of an extended organometallic network.

### 2.3. Catalysis

#### 2.3.1. Design of the Tandem Catalytic System

**Figure 5:**
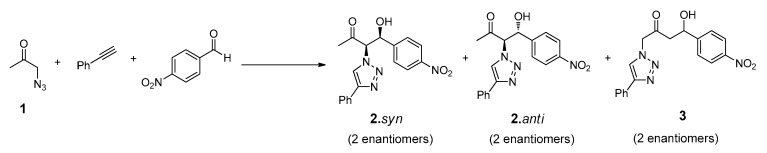
Structures of reactants and possible products involved in the tandem ‘click’-aldol reaction system.

The design of the multicomponent catalytic system requires the presence of, at least, one bifunctional compound that could undergo both ‘click’ and aldol addition reaction. The synthesis of this reactant (**N_3_Cet**) was achieved by simple reaction of the chloroacetone with sodium azide, as reported elsewhere [46]. The compounds chosen to complete the tandem system were the commercially available phenylacetylene and *p*-nitrobenzaldehyde (Figure 5). 

As usual in aldol addition reactions, two possible diastereoisomers can be obtained as main reaction products such as **2.***syn* and **2.***anti* in both antipodal forms: (*R*, *R*), (*S*, *S*) and (*R*, *S*), (*S*, *R*) for *syn* and *anti* diastereoisomers, respectively. Considering the stronger acidity of the α-hydrogen positioned between the azide and carbonyl group, the formation of the compound **3**, arising from a possible addition on the methyl group of reactant **1**, would not be expected. Preliminary experiments performed on these reactions confirmed the absence of NMR signals corresponding to this product.

**Figure 6:**
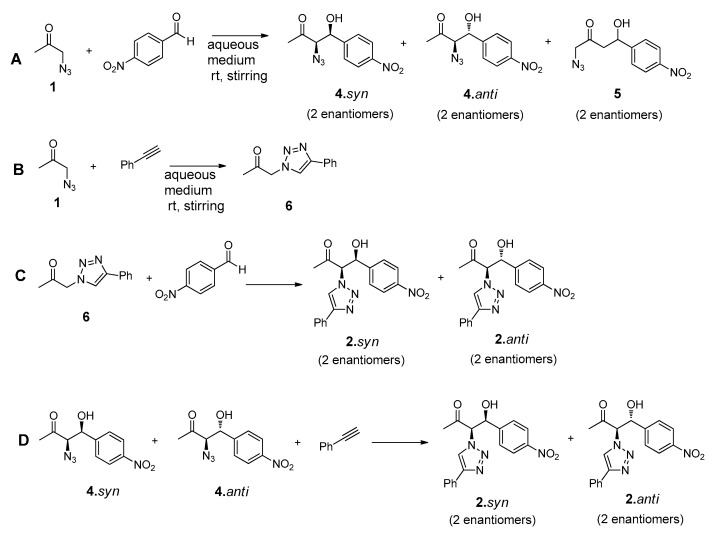
Click and aldol reactions guiding to the formation of the possible intermediates **4** and **6** and final products **2.***syn* and **2.***anti*.

This complex reaction system can be divided in four stepwise ‘click’ and aldol addition reactions, as represented in Figure 6.

The formation of the intermediate products **4.***syn* and **4.***anti* or **6** will highly depend on the competitiveness of the three-component reaction system. These intermediate products will further undergo ‘click’ or aldol addition, yielding the final reaction products (Figure 6).

#### 2.3.2. Catalysis of the Tandem ‘Click’-Aldol Reaction System

**Figure 7:**
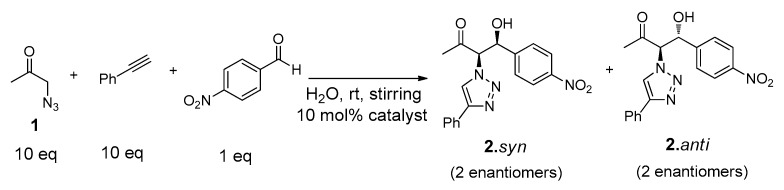
Structures of reactants and products involved in the tandem system.

The catalytic ability of the gelator Cu(I)-**PhTzVal_3_** was evaluated in the three-component system (Figure 7). The reactions were carried out in distilled water at room temperature and with stirring, for periods of four days, using 10 mol % of Cu(I)-**PhTzVal_3_** catalyst. Blank experiments in the presence of CuBr or without any catalyst were also performed.

An excess of **1** (10 eq) was used in order to avoid the occurrence of possible retro-aldol reactions. Consequently, a similar molar ratio of the commercially available phenylacetylene was also used to assure the formation of the intermediate product **6** in excess. The conversions achieved were determined by ^1^H-NMR (Table 1), where it could be observed the presence of a single diastereoisomer (see Supplementary Materials). The coupling constant between the CHN and CHOH protons determined to this product (*J* = 3.2 Hz) was similar to the one found for the same protons on the intermediate **4.***syn*, allowing its identification as **2.***syn* [46].

The obtained results highlight the good catalytic performance of the metallogel Cu(I)-**PhTzVal_3_** for both ‘click’ and aldol addition reactions, although in the former only 30% conversion has been achieved (Table 1, entry 1). However, this conversion was still considerably higher than the one obtained in the presence of pure **PhTzVal_3_** aggregates, CuBr or only in water, where no final aldol product has been detected (Table 1, entries 2–4). Although it is widely known from literature the good catalytic performance of triazole ligands in the copper(I) catalyzed Huisgen 1,3-cycloaddition [47], the conversions achieved for this reaction were relatively similar in the presence of Cu(I)-**PhTzVal_3_** or CuBr, probably due to the long reaction times (Table 1, entries 1 and 3). However, no ‘click’ product **6** has been obtained when the reaction was carried in the absence of copper (I) (Table 1, entries 2 and 4).

The catalytic behavior of the metallogel Cu(I)-**PhTzVal_3_** towards the aldol addition reaction is quite surprising, since to the best of author’s knowledge, there is no evidence in literature about the catalysis of aldol reactions in the presence of copper(I) coordinated triazole fragments. In an attempt to better understand the interesting catalytic behavior of the hydrogel Cu(I)-**PhTzVal_3_** towards the three-component system, the catalytic performance was followed for periods ranging between 8 h to 4 days (Table 2). The reactions were carried in similar conditions as the ones mentioned above and the conversions determined by ^1^H-NMR.

The results indicated in Table 2 clearly highlight the good catalytic activity of the metallogel Cu(I)-**PhTzVal_3_** towards the three-component reaction system, achieving 61% of conversion to the final product **2** just after two days of reaction in the presence 10 mol % of the metallogel catalyst (Table 2, entry 3). It is also curious to observe that the conversions achieved for **2** and **6** were relatively similar between each other during the first two days (Table 2, entry 1–2), suggesting that the **6** formed was immediately reacting with the *p*-nitrobenzaldehyde available in the reaction medium. However, it appears that there is an accentuated increase in the kinetics of the ‘click’ reaction towards its completion after two days of reaction, which coincides with the highest conversion achieved for **2**. The lower conversions achieved after three and four days of reaction, 31% and 33%, respectively, might be caused by a possible reversion of the aldol addition due to the scarce solubility of the intermediate **6** or possible instability of the final product (Table 2, entries 4–5).

The compound **2** can also be obtained if 20 eq excess of **1** is used in the three-component reaction system, in the presence of 10 mol % Cu(I)-**PhTzVal_3_** (Table 2, entry 6). Although the final product was formed in a relatively low amount (Table 2, entry 6), the conversion achieved for the intermediate product **4** (55%) was considerably higher in comparison with the one obtained for the reactions carried in the presence of 10 equivalents of **1**, where the conversion did not reach the 5% (Table 2, entries 1–5). Thus, it can be suggested that the presence of a high excess of **1** displaces the equilibrium of the aldol addition between **1** and the *p*-nitrobenzaldehyde to the products, resulting in the formation of a higher amount of **4**. Further ‘click’ coupling of the intermediate product **4.***syn* with the commercially available phenylacetylene guides to the formation of the final product **2.***syn*.

**Figure 8:**
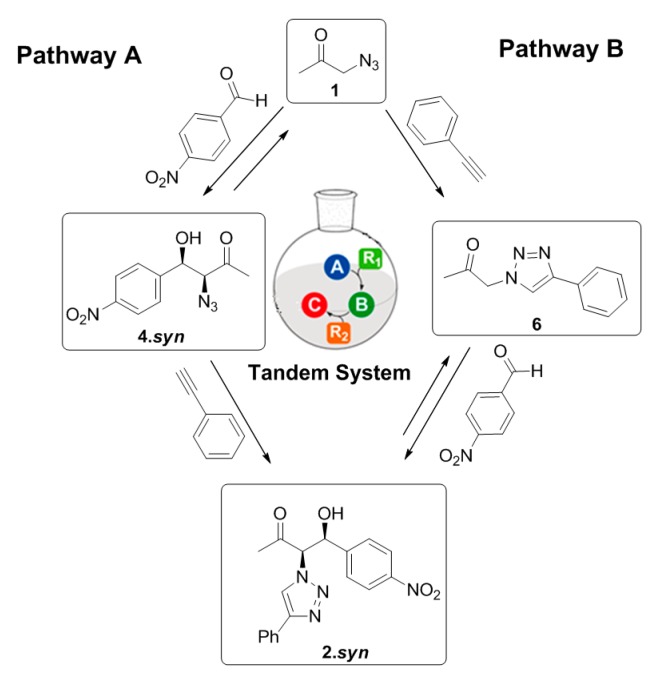
Possible reaction pathways involved in the three-component system.

This behavior suggests that the three-component reaction system is able to follow two different pathways depending on the competitiveness and on the initial reaction conditions, as represented in Figure 8.

When initial equimolar amounts of **1** and phenylacetylene are present, the three-component system follows pathway B, where the ‘click’ reaction between the **1** and the phenylacetylene is kinetically favored guiding to the formation of the intermediary **6**. This intermediate product can further undergo an aldol addition to achieve the formation of the final product **2**. However, in the presence of 20 eq. of **1**, the equilibrium of the aldol addition between **1** and the *p*-nitrobenzaldehyde is displaced and the reaction can proceed through pathway A, favoring the formation of **4**. This intermediate product can subsequently undergo a ‘click’ reaction with the phenylacetylene to achieve the final product **2**. However, the relatively similar conversion achieved for the final product (35%) through both pathways after four days’ reaction suggest that the pathway B does not favor the formation of a higher amount of final product **2** in comparison with pathway A (Table 2, entries 5–6). Nevertheless, independently on the reaction pathway followed during the multicomponent reaction system, it is clear that the gelator Cu(I)-**PhTzVal_3_** acts as a good tandem catalyst for both the aldol and ‘click’ reactions occurring in this three-component system The 55% conversion achieved in this aldol addition reaction between **1** and *p*-nitrobenzaldehyde together with the 98% conversion obtained for the ‘click’ reaction between **1** and the phenylacetylene, using 20 eq **1**, supports the good catalytic activity of the metallogel Cu(I)-**PhTzVal_3_** for both reactions (Table 2, entry 6). 

To isolate and completely characterize the final product **2**, the three-component reaction was performed in higher scale in the presence of 10 mol % Cu(I)-**PhTzVal_3_** for two days, following similar conditions as the ones used represented in Figure 7, and the resultant crude mixture purified through column chromatography using silica flash. However, the ^1^H-NMR of the resultant fractions exhibited a mixture of peaks characteristic of product degradation, which was probably caused by the acidity of the silica. This high instability of the product **2** may support the low yields obtained after four days’ reaction. Thus, the same reaction was mounted again for two days and the mixture purified by column chromatography using alumina type II as stationary phase. Also in this case, the resultant ^1^H-NMR was not completely pure, but some peaks of **2** could be detected together with characteristic degradation peaks. The presence of this product was further confirmed by high-resolution mass-spectrometry, giving a protonated adduct at *m*/*z* = 353.1249 (see Supplementary Materials). The enantiomeric excess was also determined by high pressure liquid chromatography (HPLC), suggesting that **2** is formed as a racemic mixture (see Supplementary Materials).

The mechanistic pathway by which the metallogel Cu(I)-**PhTzVal_3_** is able to catalyze this tandem three-component reaction may be similar to the one followed by type II (fuculose-1-phosphate) aldolase, where the substrate is activated by the presence of the Zn^2+^ coordinated to a histidine fragment [48]. The type II aldolases use dihydroxyacetone phosphate (DHAP) as substrate to produce 2-keto-3,4-dihydroxy adducts. It has been reported that in this reaction, the Zn^2+^ polarizes the carbonyl group of the substrate by coordination, facilitating the removal of the α-hydrogen by the glutamate residue present in the enzyme structure. In this mechanism, a tyrosine residue from the adjoining subunit also assists in the activation of the incoming aldehyde by donating a proton to stabilize the developing charge. This hydrogen bond is responsible for the asymmetric induction at C-4, while the shielding of the *Si* face of the DHAP enediolate by protein assures the correct stereochemistry of the C-3 product [49].

**Figure 9:**
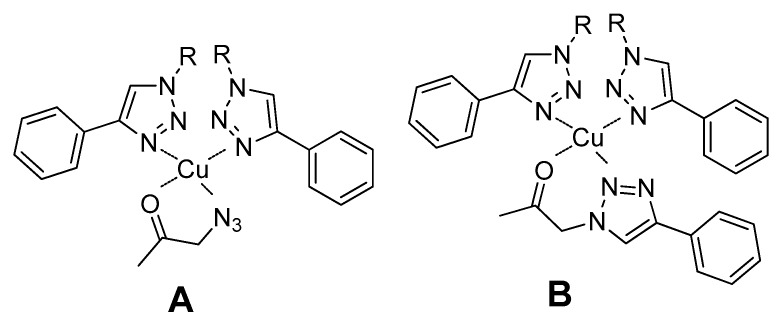
Proposed complexes formed between Cu(I) coordinated to the triazole fragments of the metallogel and the aldol addition substrate. Complex A is formed if reaction undergoes through pathway A and coordination complex B is present if reaction proceeds through pathway B.

In our specific case, the mild coordination of the triazole ligand to copper(I) may favor the coordination of the carbonyl group of the substrate of the aldol addition reaction, leading to the formation of a tetrahedral complex. Depending on the predominant reaction pathway, an azido or a triazole group of the substrate may also coordinate to the metal center (Figure 9).

**Figure 10:**
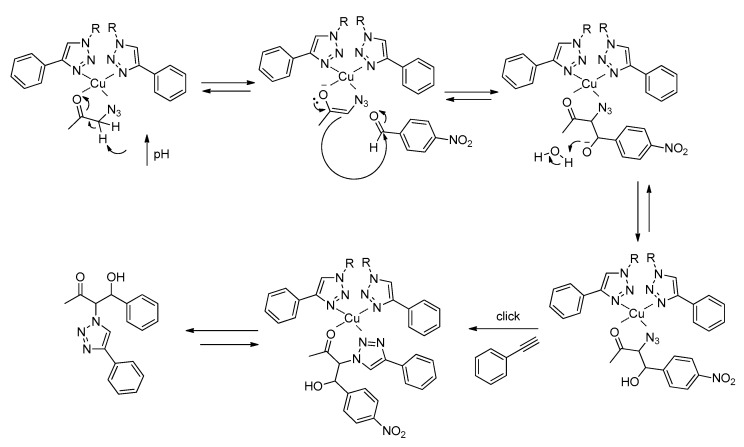
Proposed catalytic mechanism for the aldol reaction in the presence of the gel catalyst Cu(I)-**PhTzVal_3_**. The represented catalytic mechanism is illustrative of reactions following pathway A.

Similarly to type II aldolases, this coordination of the substrate to the metal center polarizes the carbonyl group, increasing the acidity of the α-methylene hydrogen. In addition, a possible increase in the basicity of the medium arising from the gelation of Cu(I)-**PhTzVal_3_** may result in the easiest removal of the α-hydrogen proton from the substrate and consequent attack on the carbonyl group of the *p*-nitrobenzaldehyde (Figure 10). Once **2** is formed, the mild coordination ability of the triazole moiety would allow the detachment of the final product from the molecular gel, making it available to embark in a new catalytic cycle.

It is important to remind that by using only CuBr as catalyst (10 mol %), no aldol product has been observed in the three-component reaction (Table 1, entry 3). This result reinforces the important role of the metallogel on the catalysis of this aldol addition reaction, suggesting that an aggregation effect may also be determinant in this catalytic activity. The change in the basicity of the medium arising from a self-assembly process has already been reported for bolaamphiphilic gels functionalized with l-proline fragments [50]. Rodríguez-Llansola *et al.* reported that such supramolecular-induced enhancement of basicity aroused from the close proximity of the pyrrolidinic residues on the surface of the gel fibers, which was suggested to favor the cooperative assistance of several basic groups on the deprotonation of the substrate and consequent formation of an intermediate enolate [19]. 

In the case of this three-component system, a small increase in the basicity of the medium provided by the formation of the Cu(I)-**PhTzVal_3_** coordination complex together with the proximity of triazole sites in the hydrogel fibres could easily catalyze the multicomponent reaction, considering the acidic nature of the α-hydrogen of **1**. The acidic environment of this proton arises not only from its position between a carbonyl and an azide group, but also from the polarization of the carbonyl group upon coordination of the substrate to the copper(I) center. Some additional contribution on the increase on the basicity of the medium due to coordination of water to the metallohydrogel complex, as reported in literature for some copper complexes, cannot be discarded [51]. This multicomponent reaction follows an auto-tandem catalytic process, since it occurs in the presence of a single catalyst and all the species are present from the beginning of the reaction. 

This tandem catalytic system join together the features of aldol addition and ‘click’ reactions, leading to the “one-pot” synthesis of a molecule containing a triazole moiety together with two adjacent stereocenters. The reported biological activities of 1,2,3-triazoles, such as anticancer, antifungal, antibacterial, antivirus, coupled with the presence of asymmetric C-C bonds potentiate the use of the resultant products as important building blocks in the synthesis of biologically active molecules [26].

## 3. Materials and Methods

### 3.1. Structural and Morphological Characterization

^1^H- and ^13^C-NMR spectra measurements were recorded in a Varian Mercury 300 MHz spectrometer (Varian, Palo Alto, CA, USA) at 30 °C. Powder X-ray diffraction (XRD) was performed at room temperature with a Bruker D4 Endeavor X-ray powder diffractometer (Bruker AXS, Madison, WI, USA) by using Cu-K radiation. A sample of the dried gel was placed on a sample holder and data were collected for 2θ values between 2 and 40° with a step size of 0.03° and a time step of 10 s. Circular dichroism (CD) spectra were recorded in a Jasco J-810 spectropolarimeter (Jasco, Peabody, MA, USA). The measurements were performed on pellets composed by 1 mg of lyophilized gel and 50 mg of KBr. For transmission electron microscopy (TEM), a small portion of the gel was placed on a nickel grid coated with carbon and allowed to dry on air. Images were recorded in a JEOL 2100 microscope (JEOL, Peabody, MA, USA). Catalytic enantiomeric excess was determined by HPLC (Agilent Technologies, Santa Clara, CA, USA) using a Chiralpack IA column, λ = 254 nm, Ethanol/Hexane (*v*/*v*: 15/85), flow rate = 1.5 mL/min.

### 3.2. Synthesis

Detailed synthetic procedures and characterisation are found in the Supplementary Materials.

### 3.3. Gelation Procedure

The gelator **PhTzVal_3_** (8 mg; 11.4 × 10^−3^ mmol) was heated in a 8 mL screw-capped vial for 5 min, in the presence of 2 mL of water. The resultant hot solution was then sonicated for 20 s, forming a swollen precipitate, which was subsequently allowed to age for 20 min at room temperature. The formation of the metallogels was achieved by heating together the gelator and the copper salt CuBr in a 2:1 molar ratio.

### 3.4. Quantification of Coordinated Copper

After the formation of the catalytic metallogel, 100 µL of surrounding solution were collected with a 1 mL syringe and filtered through a 0.22 µm filter. The filter was washed with a solution of 5% HNO_3_ (2 × 1 mL) in Milli-Q water to remove remaining uncoordinated copper. A 25 mL solution of the sample in 5% HNO_3_ was prepared and the concentration of copper(I) determined by inductively coupled plasma-mass spectrometry (ICP-MS) using an Agilent 7500 cx (Agilent Technologies, Santa Clara, CA, USA).

### 3.5. General Procedure for Catalytic Experiments

The reactant *p*-nitrobenzaldehyde (0.86 mg; 5.7 × 10^−3^ mmol) was dissolved in th desired amount of **1** and the resultant mixture added directly on a 8 mL screw-capped vial containing 2 mL of water and, when needed, 10 mol % of the desired catalyst. Phenylacetylene (5.82 mg; 5.7 × 10^−2^ mmol) was subsequently added and the mixture allowed to react at room temperature and under stirring for an adequate period of time. At the end of the experiment, the products were directly extracted with CDCl_3_ (3 × 330 µL). The organic phase was dried over anhydrous MgSO_4_ and the conversion determined by ^1^H-NMR. The catalytic experiments were performed in triplicate to assure the accuracy of the results.

## 4. Conclusions

The design and synthesis of a gelator that could be used in the catalysis of a multicomponent ‘click’-aldol reaction system was successfully achieved. The three-component reaction was performed in the presence of metallogel Cu(I)-**PhTzVal_3_** (10 mol %), achieving around 65% conversion in only two days. Furthermore, the competitiveness between the reactions in the multicomponent system could be tuned by simply adjusting the amount of reactants that are present from the outset of the reaction. The fact that both intermediates **4** and **6** could be obtained in high yields when 20 eq of **1** is present at the outset of the multicomponent reaction highlights the good catalytic activity of the metallogel Cu(I)-**PhTzVal_3_** for both reactions.

It is proposed that the catalysis of the aldol addition in the presence of the metallogel Cu(I)-**PhTzVal_3_** follows a type II aldolase mechanistic pathway, where the coordination of the substrate to the metal center results in the polarization of the carbonyl group and consequent acidification of the α-hydrogen, whose removal might be assisted by an increase in the basicity of the medium provided by the supramolecular gelation of the catalyst. 

This unexpected biomimetic catalytic activity shown by the metallogel Cu(I)-**PhTzVal_3_** may open a new insight on the application of triazole functionalized compounds on the catalysis of aldol-type reactions, as well as on the tandem catalysis of similar ‘click’-aldol multicomponent systems. Furthermore, the amino acid derived nature of this tandem metallohydrogel catalyst may confer it with additional biocompatibility and biodegradability, allowing its use in similar tandem reactions occurring in a biological environment.

## Figures and Tables

**Table 1:** Conversions achieved for the three-component reaction system after four days ^1^.

Entry	Catalyst (10 mol %)	4.*syn* (%)	2.*syn* (%)	6 (%) ^2^
1	Cu(I)-PhTzVal_3_	<5	33	97
2	PhTzVal_3_	0	0	0
3	CuBr	0	0	96
4	-	0	0	0

^1^ The reactant *p*-nitrobenzaldehyde (0.86 mg; 5.7 × 10^−3^ mmol) was dissolved in **1** (5.6 mg; 5.7 × 10^−2^ mmol) and the resultant mixture transferred to a 8 mL screw-capped vial containing 2 mL of water and, if desired, 10 mol % of catalyst. Phenylacetylene (5.82 mg; 5.7 × 10^−2^ mmol) was subsequently added and the mixture allowed to react at room temperature and under stirring for four days. At the end of the experiment, the products were directly extracted with CDCl_3_ (3 × 330 µL). The organic phase was dried over anhydrous MgSO_4_ and the conversion determined by ^1^H-NMR. ^2^ The conversion of **6** was calculated based on the phenylacetylene, while the conversion of **4** and **2** was determined considering the *p*-nitrobenzaldehyde as the limiting reagent.

**Table 2:** Conversions achieved for the three-component reaction system in the presence of Cu(I)-**PhTzVal_3_** ^1^.

Entry	Reaction Time	Reactants Proportion (*p*-Nitrobenzaldehyde:1:Phenylacetylene)	4.*syn* (%)	2.*syn* (%)	6 (%) ^2^
1	8 h	1:10:10	<5	20	13
2	1 day	1:10:10	<5	26	19
3	2 days	1:10:10	<5	61	95
4	3 days	1:10:10	<5	31	97
5	4 days	1:10:10	<5	33	97
6	4 days	1:20:10	55	35	97

^1^ Typical procedure: The reactant *p*-nitrobenzaldehyde (0.86 mg; 5.7 × 10^−3^ mmol) was dissolved in the desired amount of **1** and the resultant mixture transferred to a 8 mL screw-capped vial containing 3 mL of water and 10 mol % of Cu(I)-**PhTzVal_3_** catalyst, composed by the gelator **PhTzVal_3_** (8 mg; 11.4 × 10^−3^ mmol) and the copper salt CuBr (0.82 mg; 5.7 × 10^−3^ mmol). Phenylacetylene (5.82 mg; 5.7 × 10^−2^ mmol) was subsequently added and the mixture allowed to react at room temperature and under stirring for four days. At the end of the experiment, the products were directly extracted with CDCl_3_ (3 × 330 µL). The organic phase was dried over anhydrous MgSO_4_ and the conversion determined by ^1^H-NMR. ^2^ The conversion of **6** was calculated based on the phenylacetylene, while the conversion of **4** and **2** was determined considering the *p*-nitrobenzaldehyde as the limiting reagent.

## Supplementary Materials

Supplementary materials can be accessed at: http://www.mdpi.com/1420-3049/21/6/744/s1.

## Abbreviations

The following abbreviations are used in this manuscript:

ICP-MS: Inductively Coupled Plasma-Mass Spectrometry
WAXD: Wide-Angle X-Ray Diffraction
CD: Circular Dichroism
TEM: Transmission Electron Microscopy
Eq: Equivalents
NMR: Nuclear Magnetic Resonance
HPLC: High-Performance Liquid Chromatography

## References

[1] Ricca E., Brucher B. (2011). Multi-enzymatic cascade reactions: Overview and Perspectives. Adv. Synth. Catal..

[2] Clement M., Corma A., Iborra S., Sabater M. (2014). Heterogeneous catalysts for tandem reactions. ACS Catal..

[3] Liu J., Chen L., Cui H., Zhang J., Zhang L., Su C. (2014). Applications of metal-organic frameworks in heterogeneous supramolecular catalysis. Chem. Soc. Rev..

[4] Yuan Z., Chen J., Zeng Y., Li Y., Han Y., Li Y. (2011). Unsurpassed cage effect for photolysis of dibenzyl ketones in water-soluble dendrimers. Org. Biomol. Chem..

[5] Astruc D., Boisselier E., Ornelas C. (2010). Dendrimers designed for functions: From physical, photophysical, and supramolecular properties to applications in sensing, catalysis, molecular electronics, photonics and nanomedicine. Chem. Rev..

[6] Raynal M., Ballester P., Vidal-Ferran A., van Leeuwen P. (2014). Supramolecular catalysis. Part 1: Non-covalent interactions as a tool for building and modifying homogeneous catalysts. Chem. Soc. Rev..

[7] Meeuwissen J., Reek N.H. (2010). Supramolecular catalysis beyond enzyme mimics. Nat. Chem..

[8] Raynal M., Ballester P., Vidal-Ferran A., van Leeuwen P. (2014). Supramolecular catalysis. Part 2: Artificial enzyme mimics. Chem. Soc. Rev..

[9] Yuan Z., Zheng S., Zeng Y., Chen J., Han Y., Li Y., Li Y. (2010). Photosensitized oxidation of alkenes with dendrimers as microreactors: Controllable selectivity between energy and electron transfer pathway. New J. Chem..

[10] Yuan Z., Liang F. (2014). Photoreactions in amphiphilic microreactors with “soft” cavities: Controlling product selectivity in solution. Curr. Org. Chem..

[11] Robert C., Thomas C. (2013). Tandem catalysis: A new approach to polymers. Chem. Soc. Rev..

[12] Lohr T., Marks T. (2015). Orthogonal tandem catalysis. Nat. Chem..

[13] Fogg D., Santos E. (2004). Tandem catalysis: A taxonomy and illustrative review. Coord. Chem. Rev..

[14] Wasilke J., Stephen J., Obrey J., Baker T., Bazan G. (2005). Concurrent tandem catalysis. Chem. Rev..

[15] Ramarchary D., Barbas III C. (2005). Towards organo-click chemistry: Development of organocatalytic multicomponent reactions through combinations of aldol, Wittig, Knoevenagel, Michael, Diels-Alder and Huisgen cycloaddition reactions. Chem. Eur. J..

[16] Nelson S. (1998). Catalyzed enantioselective aldol additions of latent enolate equivalents. Tetrahedron Asymmetry.

[17] List B., Lerner R., Barbas III C. (2000). Proline-catalyzed direct asymmetric aldol reactions. J. Am. Chem. Soc..

[18] Sakthivel K., Notz W., Bui T., Barbas III C. (2001). Amino acid catalyzed direct asymmetric aldol reactions: A bioorganic approach to catalytic asymmetric carbon-carbon bond-forming reactions. J. Am. Chem. Soc..

[19] Rodríguez-Llansola F., Escuder B., Miravet J. (2009). Switchable performance of an l-proline-derived basic catalyst controlled by supramolecular gelation. JACS.

[20] Rodríguez-Llansola F., Miravet J., Escuder B. (2009). A supramolecular hydrogel as a reusable heterogeneous catalyst for the direct aldol reaction. Chem. Commun..

[21] Wang X., Meng Q., Perl N., Xu Y., Leighton J. (2005). Tandem aldol-allylation and aldol-aldol reactions with ketone-derived enolsilanes: Highly diastereoselective single-step synthesis of complex teriary carbinols. J. Am. Chem. Soc..

[22] Arnold L., Naasz R., Minnaard A., Feringa B. (2002). Catalytic enantioselective synthesis of (−)-Prostaglandin E_1_ methyl ester based on a tandem 1,4-addition-aldol reaction. J. Org. Chem..

[23] Feringa B., Pineschi M., Arnold L., Imbos R., de Vries A. (1997). Highly enantioselective catalytic conjugate addition and tandem-conjugate addition-aldol reactions of organizing reagents. Angew. Chem. Int. Ed. Eng..

[24] Bui T., Barbas C. (2000). A proline-catalyzed asymmetric Robinson annulation reaction. Tetrahedron Lett..

[25] Vhowdari N., Ramachary D., Barbas III C. (2003). Organocatalytic asymmetric assembly reactions: One-pot synthesis of functionalized β-amino alcohols from aldehydes, ketones and azidocarboxylates. Org. Lett..

[26] Hou J., Liu X., Shen J., Zhao G., Wang P. (2012). The impact of ‘click’ chemistry in medicinal chemistry. Expert Opin. Drug Discov..

[27] Moses J., Moorhouse A. (2007). The growing applications of click chemistry. Chem. Soc. Rev..

[28] Tornoe C., Christensen C., Meldal M. (2002). Peptidotriazoles on solid phase: [1,2,3]-triazoles by regiospecific copper(I)-catalyzed 1,3-dipolar cycloadditions of terminal alkynes to azides. J. Org. Chem..

[29] Rostovtsev V., Green L., Fokin V., Sharpless B. (2002). A stepwise Huisgen cycloaddition process: Copper(I)-catalyzed regioselective “ligation” of azides and terminal alkynes. Angew. Chem. Int. Ed..

[30] Araújo M., Díaz-Oltra S., Escuder B. (2016). Triazolyl-based molecular gels as ligands for autocatalytic ‘click’ reactions. Chem. Eur. J..

[31] Odlo K., Hoydahl E., Hansen T. (2007). One-pot synthesis of 1,4-disubstituted 1,2,3-triazoles from terminal acetylenes and *in situ* generated azides. Tetrahedron Lett..

[32] Appukkuttan P., Dehaen W., Fokin V., der Eycken E. (2004). A microwave-assisted click chemistry synthesis of 1,4-disubstituted 1,2,3-triazoles via a copper(I)-catalyzed three-component reaction. Org. Lett..

[33] Campbell-Verduyn L., Szymanski W., Postema C., Dierckx R., Elsinga P., Janssen D., Feringa B. (2010). One pot ‘click’ reactions: Tandem enantioselective biocatalytic epoxide ring opening and [3+2] azide alkyne cycloaddition. Chem. Commun..

[34] Binder W., Sachsenhofer R. (2008). ‘Click’ Chemistry in polymer and material science: An update. Macromol. Rapid Commun..

[35] Mlynarski J., Paadowska J. (2008). Catalytic asymmetric aldol reactions in aqueous media. Chem. Soc. Rev..

[36] Becerril J., Bolte M., Burguete M., Galindo F., Garcia-España E., Luis S., Miravet J. (2003). Efficient macrocyclization of u-turn preorganized peptidomimetics: The role of intramolecular H-bond and solvophobic effects. J. Am. Chem. Soc..

[37] Jin Q., Zhang L., Cao H., Wang T., Zhu X., Jiang J., Liu M. (2011). Self-assembly of copper(II) ion-mediated nanotube and its supramolecular chiral catalytic behavior. Langmuir.

[38] He Y., Bian Z., Kang C., Cheng Y., Gao L. (2010). Chiral binaphthylbisbipyridine-based copper(I) coordination polymer gels as supramolecular catalysts. Chem. Commun..

[39] Bunzen J., Bruhn T., Bringmann G., Lützen A. (2009). Synthesis and helicate formation of a new family of BINOL-based bis(bipyridine) ligands. J. Am. Chem. Soc..

[40] Lam S., Yam V. (2010). Synthesis, characterisation and photophysical study of alkynylrhenium(I) tricarbonyl diamine complexes and their metal-ion coordination-assisted metallogelation properties. Chem. Eur. J..

[41] Shen J., Mao G., Zhou Y., Jiang Y., Zhang H. (2010). A ligand-chirality controlled supramolecular hydrogel. Dalton Trans..

[42] Joshi S., Kulkarni N. (2009). A new trinuclear Cu(II) complex of inositol as a hydrogelator. Chem. Commun..

[43] Chan T., Hilgraf R., Sharpless B., Fokin V. (2004). Polytriazoles as copper(I)-stabilizing ligands in catalysis. Org. Lett..

[44] Díez-González S. (2011). Well-defined copper(I) complexes for Click azide-alkyne cycloaddition reactions: One Click beyond. Catal. Sci. Technol..

[45] Yu G., Yan X., Chengyou H., Huang F. (2013). Characterization of supramolecular gels. Chem. Soc. Rev..

[46] Martinez-Castañeda A., Kedziora K., Lavandera I., Rodríguez-Solla H., Concellón C., del Amo V. (2014). Highly enantioselective synthesis of α-azido-β-hydroxy methyl ketones catalyzed by a cooperative proline-guanidinium salt system. Chem. Commun..

[47] Schulze B., Schubert U. (2014). Beyond click chemistry—Supramolecular interactions of 1,2,3-triazoles. Chem. Soc. Rev..

[48] Machajewski T., Wong C. (2000). The catalytic asymmetric aldol reaction. Angew. Chem. Int. Ed..

[49] Fessner W., Schneider A., Held H., Sinerius G., Walter C., Hixon M., Schloss J. (1996). The mechanism of class II, metal-dependent aldolases. Angew. Chem. Int. Ed. Eng..

[50] Rodríguez-Llansola F., Escuder B., Miravet J. (2009). Remarkable increase in basicity associated with supramolecular gelation. Org. Biomol. Chem..

[51] Zhang X., Liu X., Philips D.L., Zhao C. (2016). Mechanistic insights into the factors that influence the DNA nuclease activity of mononuclear facial copper complexes containing hetero-substituted cyclens. ACS Catal..

