# Supplementary material for: Tandem Catalysis of an Aldol-‘Click’ Reaction System within a Molecular Hydrogel
Source: Molecules. 2016 Jun 8;21(6):744. doi: 10.3390/molecules21060744 (PMC6273958; doi:10.3390/molecules21060744)
Supplement: Supplementary file 1 [file molecules-21-00744-s001.pdf]

Supplementary Materials: Tandem Catalysis of an Aldol-'Click' Reaction System within a Molecular Gel

Marco Araújo, Iván Muñoz Capdevila, Santiago Díaz-Oltra and Beatriu Escuder

1. Materials

4-Bromobutyric acid, *N,N′*-Dicyclohexylcarbodiimide, *N*-hydroxysuccinimide, sodium azide, 4-pentynoic acid and Phenylacetylene were purchased on Acros Organics and used as received.

2. Synthesis of the Phenyltriazole Derivative Compounds

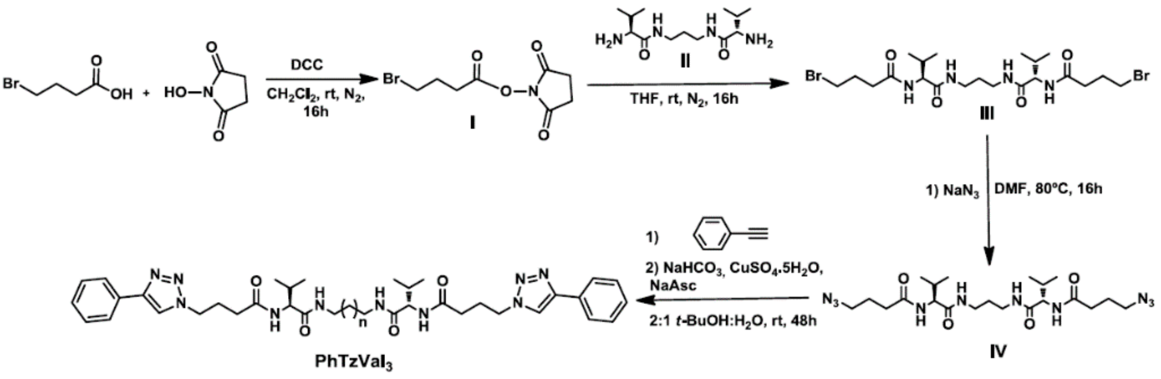

Figure S1. Scheme for the synthesis of **PhtzVal**$_3$ gelators.

2.1. *Synthesis of Compound* I

A solution of *N,N*-dicyclohexylcarbodiimide (6.240 g, 30.24 mmol) in anhydrous CH_2Cl_2 (15 mL) was added dropwise to a suspension of bromobutyric acid (5 g, 29.94 mmol) and *N*-hydroxysuccinimide (3.446 g, 29.94 mmol) in CH_2Cl_2 (30 mL) under N_2 at 0 °C. After stirring overnight between at rt, the white precipitate was filtered and the resultant solution concentrated to half and put into the freezer (−20 °C) overnight. The remaining dicyclohexylurea was filtered again and the resultant solution evaporated, giving a pale brown solid (4.060 g, 70%). Characterization: ^{1}H-NMR (300 MHz, DMSO): δ (ppm) 3.59 (t, *J* = 6.8 Hz, $BrC\underline{H}_2CH_2$-, 2H), 2.85–2.81 (m, $C\underline{H}_2CONCOC\underline{H}_2C\underline{H}_2$-, 6H), 2.15 (q, *J* = 6.8 Hz, $BrCH_2C\underline{H}_2$-, 2H). ^{13}C-NMR (75 MHz, $CDCl_3$): δ (ppm) 169.00, 167.75, 31.48, 29.48, 27.45, 25.57.

2.2. *Synthesis of Compound* II

Compound **II** was synthesized as previously reported [1].

2.3. *Synthesis of Compound* III

A solution of **I** (1 g, 3.67 mmol) in THF (15 mL) was added dropwise to a solution of **II** (2.133 g, 8.08 mmol) in THF (10 mL) under N_2 at rt. After stirring for 2 h, the resultant precipitate was filtered, washed with 0.1 M NaOH (10 mL) and water (20 mL), and dried under vacuum, affording **III** as a white solid. The compound was directly used in the next step without further purification.

2.4. *Synthesis of Compound IV*

A solution of compound **III** (0.860 g, 1.508 mmol) in DMF (10 mL) was added to a suspension of NaN_3 (0.980 g, 15.08 mmol) in DMF (10 mL) and heated under N_2 to 80 °C overnight. After cooling to rt, the crude was poured into cold water and the resultant precipitate filtered, washed with water (10 mL) and dried under vacuum, obtaining $N_3Val_3N_3$ as a bright brown solid (0.425 g; 57%). Characterization: ^{1}H-NMR (300 MHz, DMSO): δ (ppm) 7.87 (m, -CON$\underline{H}$CH + -CON$\underline{H}$$CH_2$, 2H), 4.05

(**t**, *J* = 7.9 Hz, -CONHC$\underline{H}$, 1H), 3.27 (m, $N_3C\underline{H_2}CH_2CH_2$-, 2H), 3.10 (m, -CONHC$\underline{H_2}$, 2H), 2.23 (m, $N_3CH_2C\underline{H_2}CH_2$-, 2H), 1.92 (m, -C$\underline{H}$($CH_3$)$_2$, 1H), 1.73 (m, $N_3CH_2C\underline{H_2}CH_2$-, 2H), 1.50 (m, -$CH_2C\underline{H_2}$, 2H), 0.82 (d, *J* = 6.7 Hz, -CH(C$\underline{H_3}$)$_2$, 6H). ^{13}C-NMR (75 MHz, DMSO): δ (ppm) 171.81, 171.42, 58.40, 50.77, 36.65, 32.46, 30.71, 29.55, 25.04, 19.64, 18.09. ESI-MS (*m*/*z*) = 495.3152 $[M + H]^+$; $C_{21}H_{38}N_{10}O_4$. Calculated for $C_{21}H_{38}N_{10}O_4$: 491.3156.

2.5. *Synthesis of Compound* **PhTzVal$_3$**

Phenylacetylene (233 μL, 2.12 mmol) was added to a suspension of **IV** (0.350 g, 0.71 mmol) in mixture of 2:1 *t*-BuOH:H_2O (15 mL), followed by the addition of sodium hydrogenocarbonate (0.019 g, 0.226 mmol), $CuSO_4{\cdot}5H_2O$ (0.027 g, 0.106 mmol, 5 mol%) and sodium ascorbate (0.084 g, 0.425 mmol). After stirring at room temperature for 48 h, *t*-BuOH was evaporated and treated with 1 M HCl (10 mL). The resultant precipitate was filtered under vacuum and washed with water (3 × 10 mL) and diethylether (1 × 10 mL). The product was filtered through silica (90:10 $CHCl_3$:MeOH) to remove possible coordinated copper, giving PhTzVal$_3$ as a pale yellow solid (0.346 g; 70%). Characterization: ^{1}H-NMR (300 MHz, DMSO): δ (ppm) 8.55 (s, triazole-$\underline{H}$, 1H), 7.86 (m, Ph-$\underline{H}$ + -CON$\underline{H}$CH + CON$\underline{H}$CH$_2$, 4H), 7.43 (t, *J* = 7.4 Hz, Ph-$\underline{H}$, 2H), 7.31 (t, *J* = 7.4 Hz, Ph-$\underline{H}$, 1H), 4.38 (t, *J* = 6.6 Hz, Triazole-C$\underline{H_2}$CH$_2$CH$_2$-, 2H), 4.06 (t, *J* = 7.8 Hz, -CONHC$\underline{H}$, 1H), 3.4 (m, -CONHC$\underline{H_2}$, 2H), 2.21 (m, Triazole-CH$_2$CH$_2$C$\underline{H_2}$-, 2H), 2.07 (m, Triazole-CH$_2$CH$_2$CH$_2$-, 2H), 1.91 (m, C$\underline{H}$C(CH_3)$_2$, 1H), 1.51 (m, -CH$_2$C$\underline{H_2}$, 1H), 0.81 (d, *J* = 6.4 Hz, -CH(C$\underline{H_3}$)$_2$, 6H). ^{13}C (75 MHz, DMSO): δ (ppm) 171.64, 171.43, 146.77, 131.31, 129.30, 128.22, 125.57, 121.79, 58.44, 49.57, 36.69, 32.31, 29.58, 26.35, 19.66, 18.68. ESI-MS (*m*/*z*) = 721.3912 $[M + Na]^+$; $C_{37}H_{50}N_{10}O_4$. Calculated for $C_{37}H_{50}N_{10}O_4$: 721.3914.

2.6. *Synthesis of Compound* **1**

Compound **1** was synthesized as previously reported [2].

2.7. *Synthesis of Compound* **6**

Acetoazide (0.2 g; 2.03 mmol; 0.181 mL) and phenylacetylene (0.228 g; 2.23 mmol; 0.245 mL) were dissolved in 1:1 *t*-BuOH:H_2O (4 mL). To this solution, $CuSO_4{\cdot}5H_2O$ (0.050 g; 0.203 mmol) and sodium ascorbate (0.161 g; 0.812 mmol) were added, and the mixture stirred for 16 h at room temperature. EtOAc (20 mL) was added to the crude product and the organic phase washed with a saturated solution of $NaHCO_3$ (3 × 5 mL) and brine (3 × 5 mL). The organic extracts were dried over anhydrous $MgSO_4$ and the solvents removed on the rotavapor to give a pale yellow solid (0.31 g; 76%). Characterization: ^{1}H-NMR (300 MHz, $CDCl_3$): δ (ppm) 7.82 (m, triazole-$\underline{H}$ + Ph-$\underline{H}$, 3H), 7.35 (m, Ph-$\underline{H}$ +, 3H), 5.23 (s, -COC$\underline{H_2}$, 2H), 2.26 (s, C$\underline{H_3}$CO-, 3H). ^{13}C-NMR (75 MHz, $CDCl_3$): δ (ppm) 199.03, 148.29, 130.29, 128.83, 125.80, 120.95, 58.53, 27.21. ESI-MS (*m*/*z*) = 202.0981 $[M + H]^+$; $C_{11}H_{11}N_3O$. Calculated for $C_{11}H_{11}N_3O$: 202.0980.

3. ^{1}H- and ^{13}C-NMR Spectra

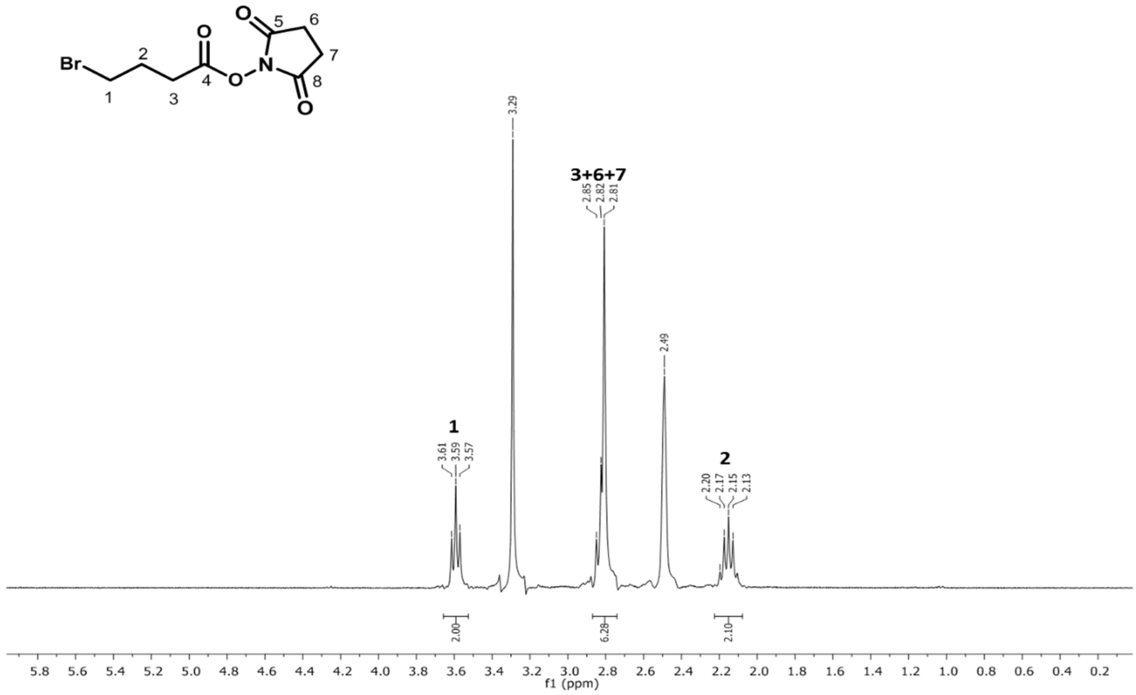

Figure S2. ^{1}H-NMR spectrum of compound **I** (DMSO-d_6, 300 MHz).

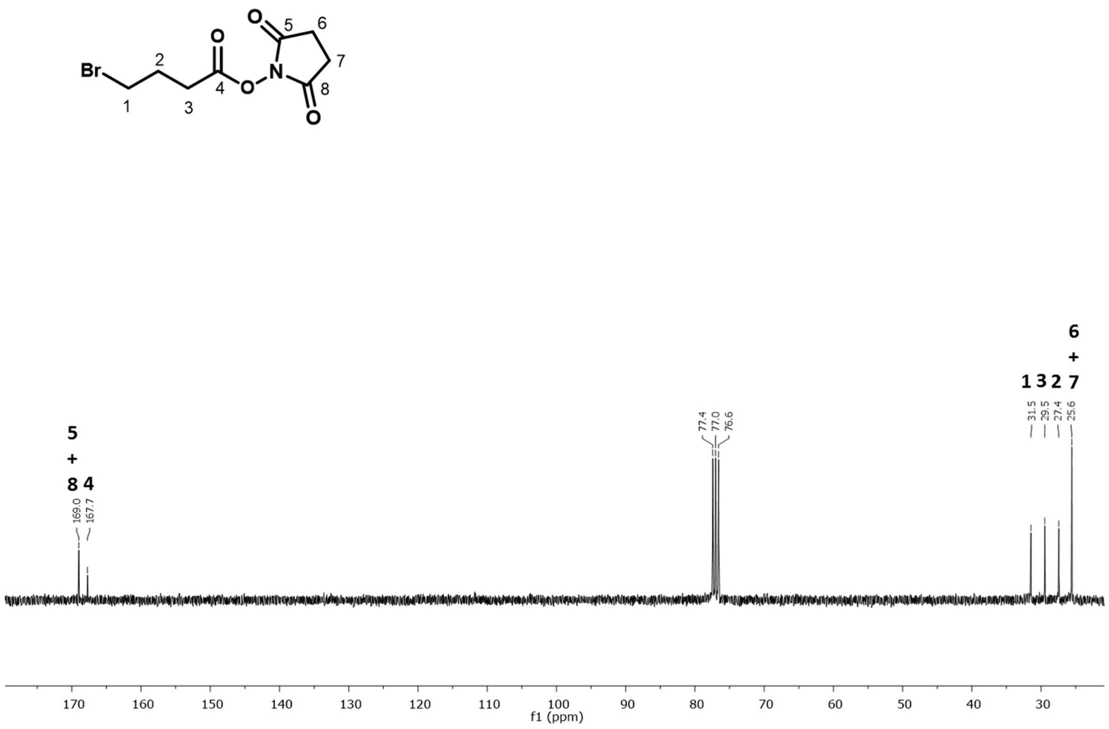

Figure S3. ^{13}C-NMR spectrum of compound **I** ($CDCl_3$, 300 MHz).

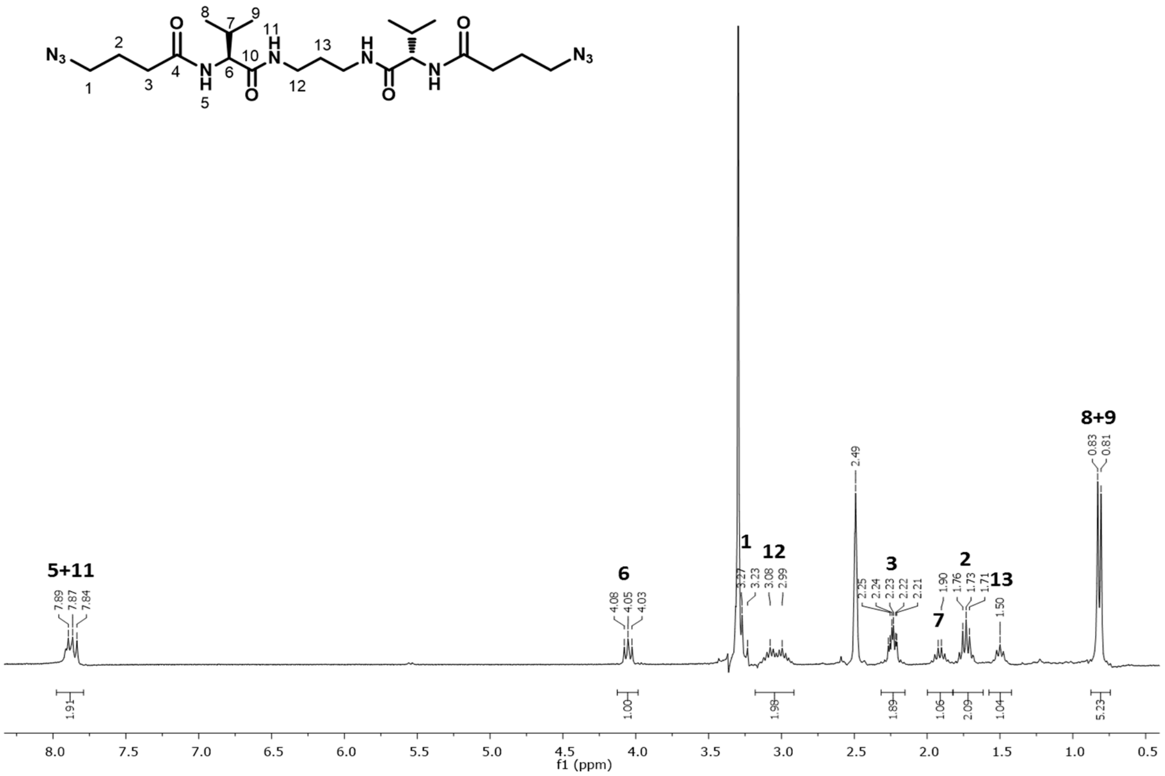

Figure S4. ^{1}H-NMR spectrum of compound **IV** (DMSO-d_6, 300 MHz).

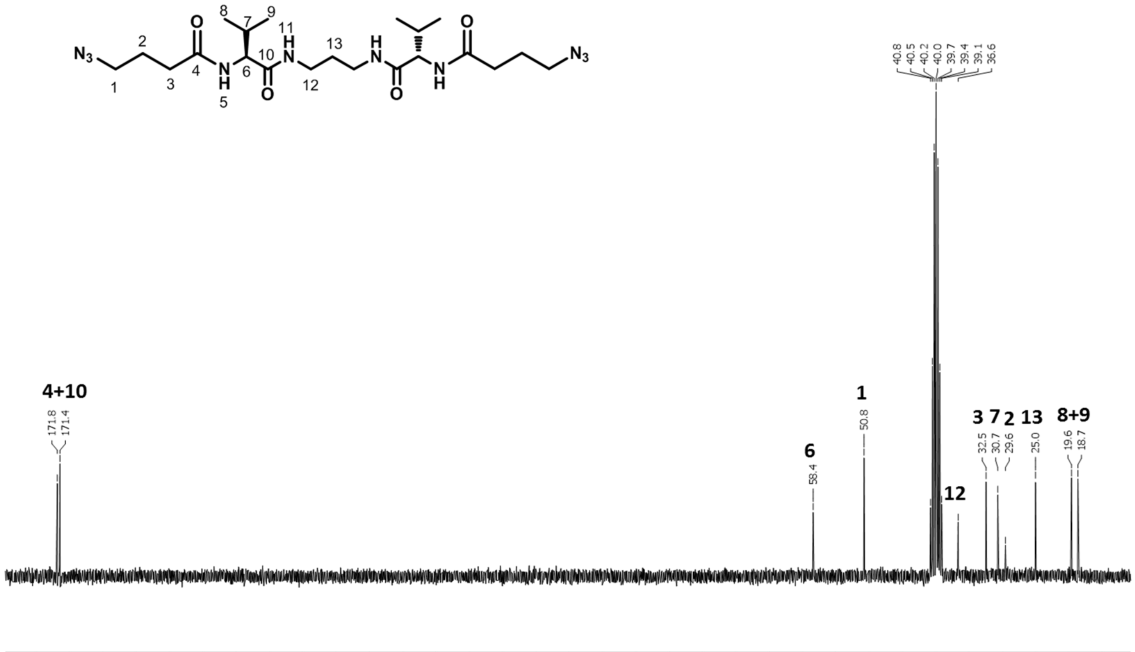

Figure S5. ^{13}C-NMR spectrum of compound **IV** (DMSO-d_6, 300 MHz).

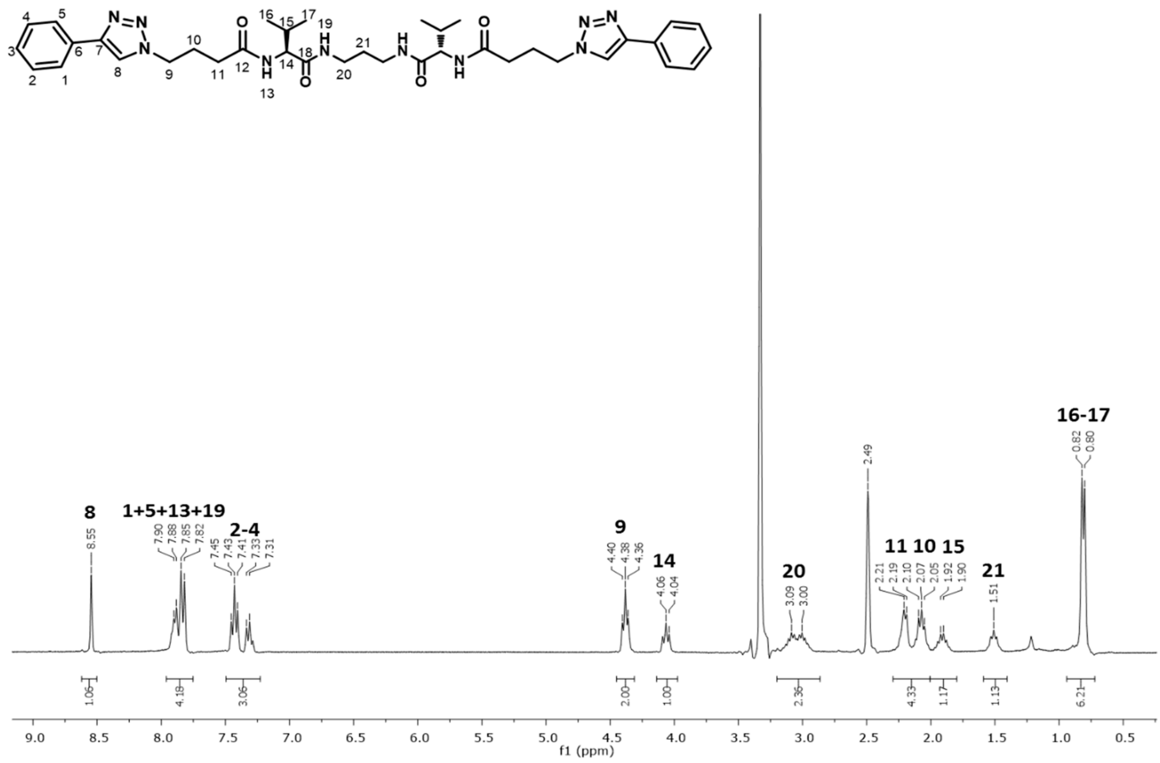

Figure S6. ^{1}H-NMR spectrum of compound **PhTzVal$_3$** (DMSO-d_6, 300 MHz).

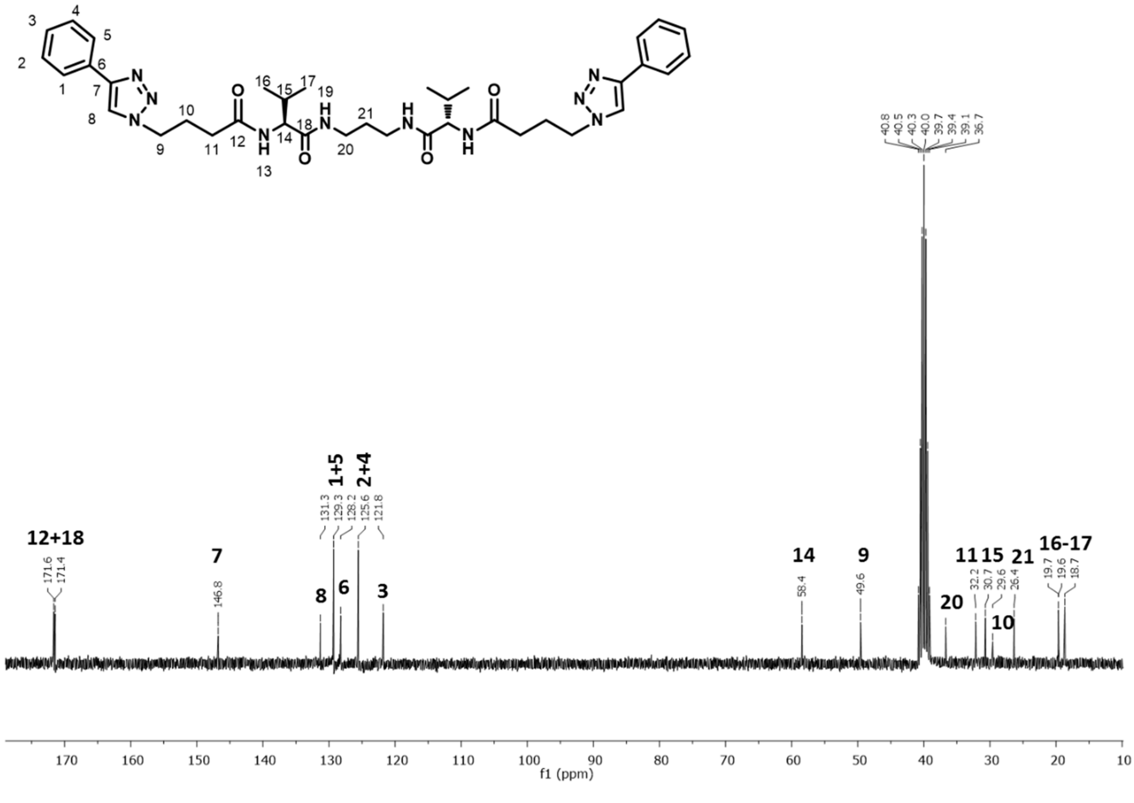

Figure S7. ^{13}C-NMR spectrum of compound **PhTzVal$_3$** (DMSO-d_6, 300 MHz).

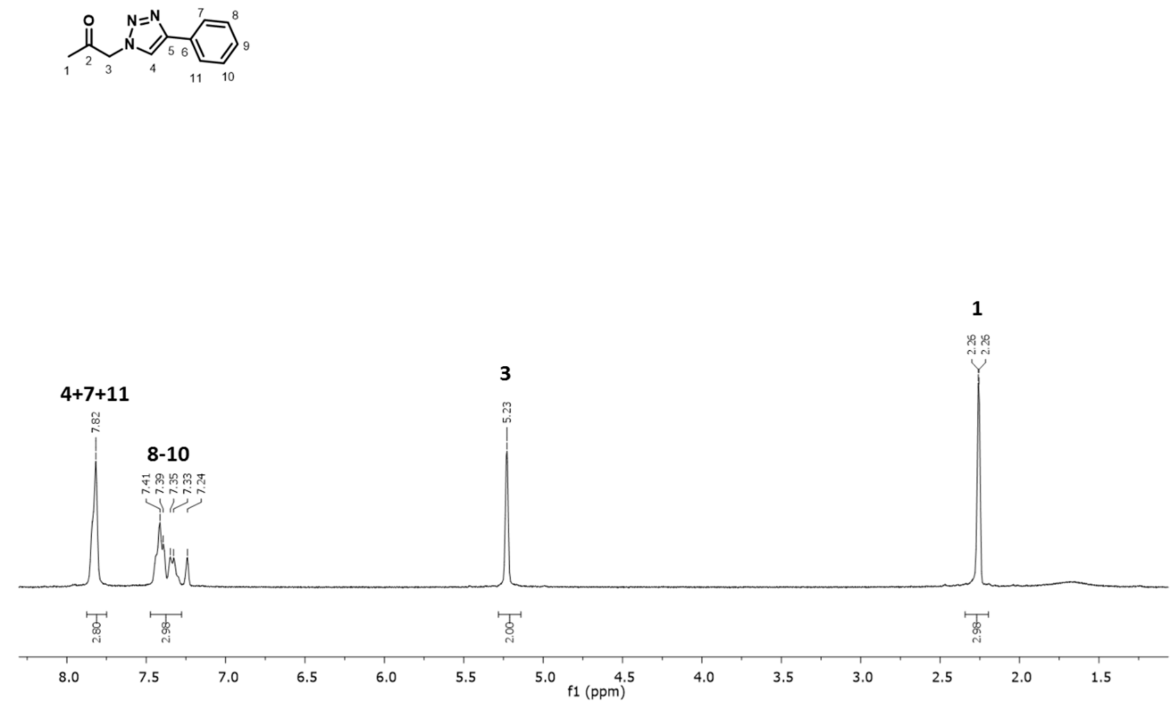

Figure S8. ^{1}H-NMR spectrum of compound **6** ($CDCl_3$, 300 MHz).

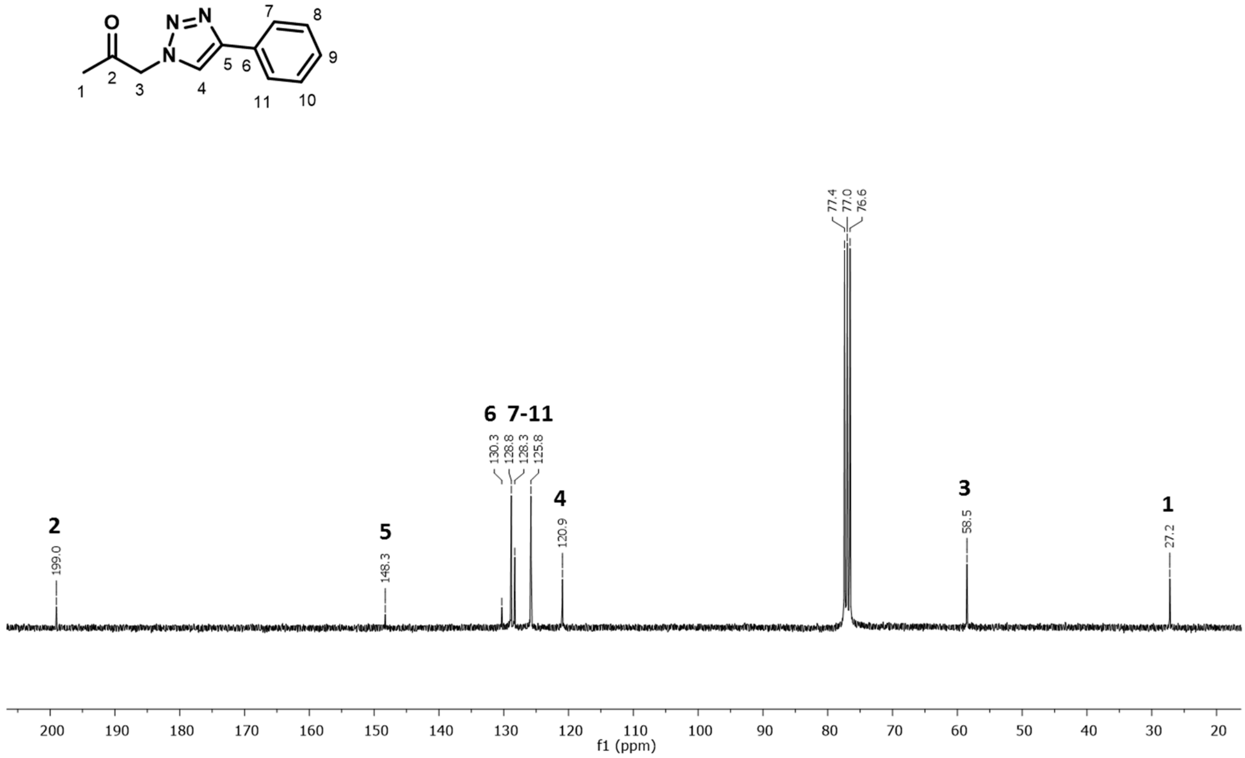

Figure S9. ^{13}C-NMR spectrum of compound **6** ($CDCl_3$, 300 MHz).

4. Typical ^{1}H-NMR Spectrum of a Catalysis

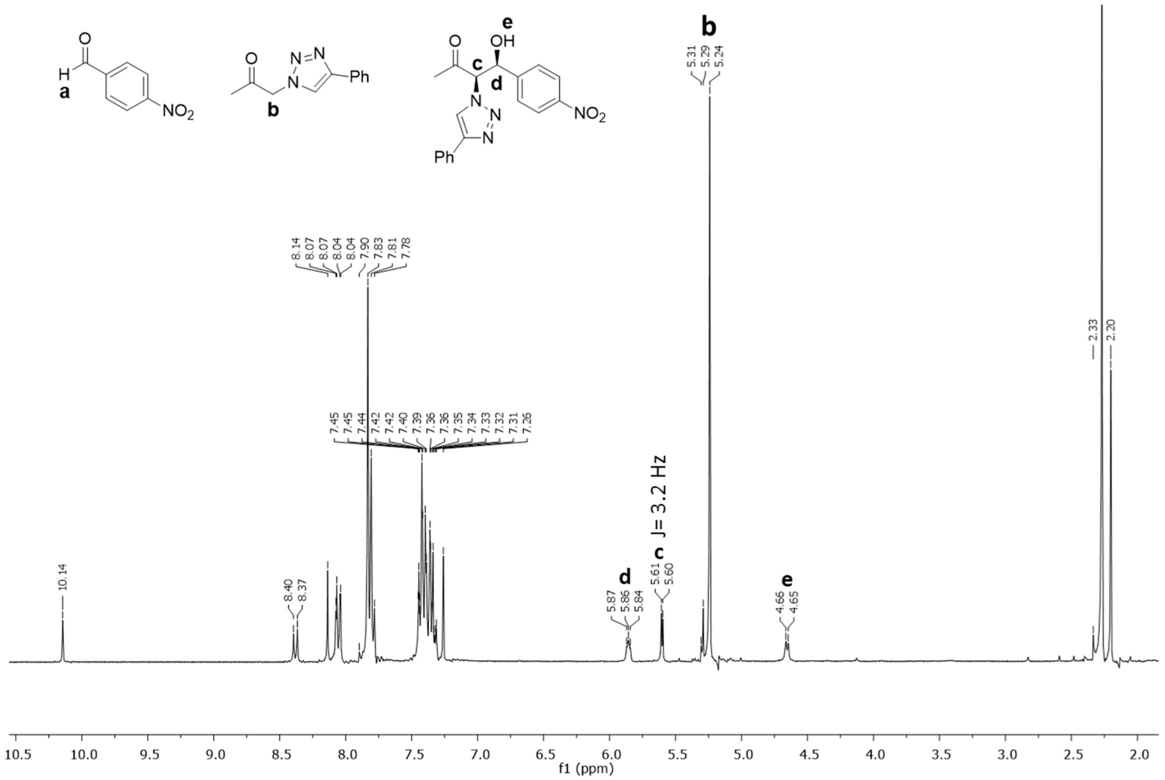

Figure S10. Typical ^{1}H-NMR spectrum of a catalytic reaction in the presence of 10 mol% Cu(I)-**PhTzVal$_3$** ($CDCl_3$, 300 MHz).

5. Mass Spectra of Final Product from the Tandem Catalytic System

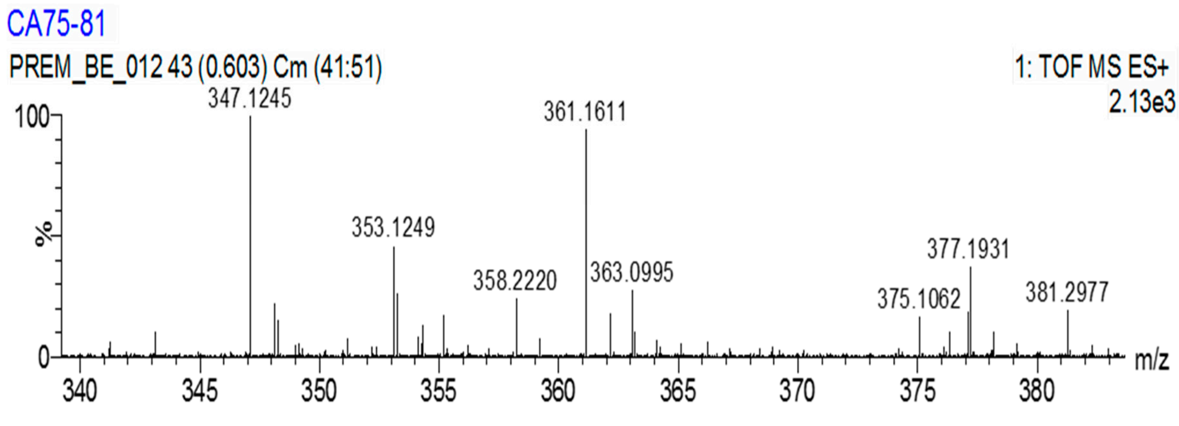

Figure S11. High-resolution mass spectrometry of the final product, isolated by type II alumina column chromatography of the crude belonging to the three component reaction carried for 2 days in the presence of 10 mol % Cu(I)-**PhTzVal$_3$**.

6. HPLC Chromatogram for the Determination of Enantiomeric Excess of the Final Product from the Tandem Catalytic System

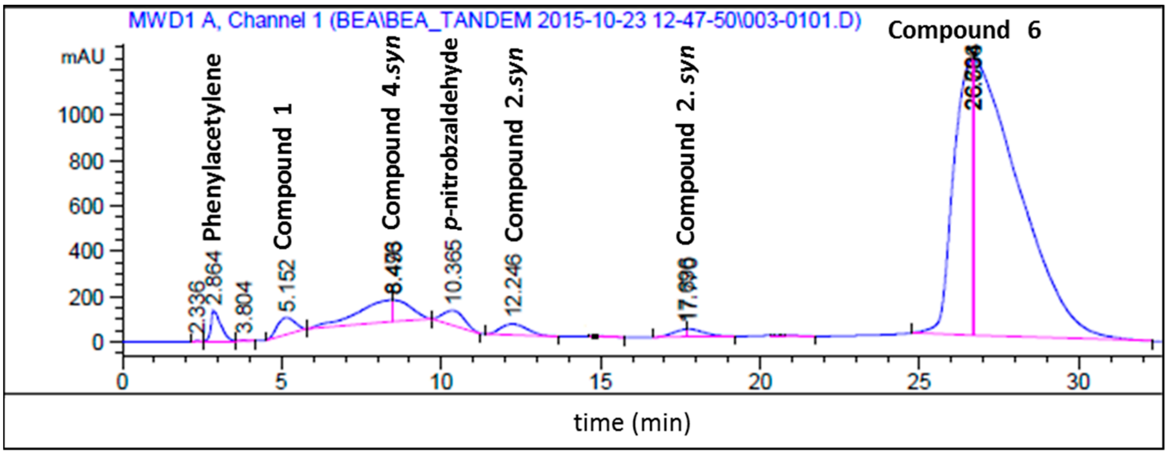

Figure S12. HPLC Chromatogram of the three-component reaction carried for 2 days in the presence of 10 mol% Cu(I)-**PhTzVal**3.

References

1. Becerril, J.; Bolte, M.; Burguete, M.; Galindo, F.; Garcia-España, E.; Luis, S.; Miravet, J. Efficient macrocyclization of u-turn preorganized peptidomimetics: The role of intramolecular H-bond and solvophobic effects. *J. Am. Chem. Soc.* **2003**, *125*, 6677–6686.
2. Martinez-Castañeda, A.; Kedziora, K.; Lavandera, I.; Rodríguez-Solla, H.; Concellón, C.; del Amo, V. Highly enantioselective synthesis of α-azido-β-hydroxy methyl ketones catalysed by a cooperative proline-guanidinium salt system. *Chem. Commun.* **2014**, *50*, 2598–2600.
